# COVID-19 double jeopardy: the overwhelming impact of the social determinants of health

**DOI:** 10.1186/s12939-022-01629-0

**Published:** 2022-05-24

**Authors:** Elizabeth Badalov, Liz Blackler, Amy E. Scharf, Konstantina Matsoukas, Sanjay Chawla, Louis P. Voigt, Arthur Kuflik

**Affiliations:** 1 City University of New York (CUNY) Hunter College, New York, NY USA; 2 Ethics Committee Memorial Sloan Kettering Cancer Center, New York, NY USA; 3 Medical Library Memorial Sloan Kettering Cancer Center, New York, NY USA; 4 Department of Anesthesiology, Pain and Critical Care Medicine Memorial Sloan Kettering Center, New York, NY USA; 5 Department of Medicine Memorial Sloan Kettering Center, New York, NY USA; 6 Department of Anesthesiology Weill Cornel Medical Center, New York, NY USA; 7 Department of Medicine Weill Cornell Medical Center, New York, NY USA; 8 Columbia University, New York, NY USA

**Keywords:** COVID-19, Pandemic, Allocation guidelines, Social determinants of health, Inequities

## Abstract

**Background:**

The COVID-19 pandemic has strained healthcare systems by creating a tragic imbalance between needs and resources. Governments and healthcare organizations have adapted to this pronounced scarcity by applying allocation guidelines to facilitate life-or-death decision-making, reduce bias, and save as many lives as possible. However, we argue that in societies beset by longstanding inequities, these approaches fall short as mortality patterns for historically discriminated against communities have been disturbingly higher than in the general population.

**Methods:**

We review attack and fatality rates; survey allocation protocols designed to deal with the extreme scarcity characteristic of the earliest phases of the pandemic; and highlight the larger ethical perspectives (Utilitarianism, non-Utilitarian Rawlsian justice) that might justify such allocation practices.

**Results:**

The COVID-19 pandemic has dramatically amplified the dire effects of disparities with respect to the social determinants of health. Patients in historically marginalized groups not only have significantly poorer health prospects but also lower prospects of accessing high quality medical care and benefitting from it even when available. Thus, mortality among minority groups has ranged from 1.9 to 2.4 times greater than the rest of the population. Standard allocation schemas, that prioritize those most likely to benefit, perpetuate and may even exacerbate preexisting systemic injustices.

**Conclusions:**

To be better prepared for the inevitable next pandemic, we must urgently begin the monumental project of addressing and reforming the structural inequities in US society that account for the strikingly disparate mortality rates we have witnessed over the course of the current pandemic.

**Supplementary Information:**

The online version contains supplementary material available at 10.1186/s12939-022-01629-0.

The COVID-19 pandemic has strained healthcare systems across the globe with overwhelming shortages in essential resources: healthcare workers, personal protective equipment (PPE), ventilators, and hospital beds [1, 2]. In the earliest phases, as the needs for medical care grew to inordinate levels, various schemes were formulated to allocate scarce medical resources, facilitate life-or-death decision-making, maximize the saving of human lives, and achieve just and equitable outcomes. In general, allocation guidelines prioritize individuals most likely to benefit from these scarce medical interventions. However, historically marginalized communities in the US – particularly Indigenous-Americans, Native Alaskans, African Americans, and Hispanic-Americans – have disproportionately suffered and died from COVID-19. But why?

We believe the best explanation has to do with entrenched inequalities – (i) most clearly perhaps, unequal access to quality healthcare itself given, for example, limited or non-existent health insurance; greater distance to hospitals; and fewer physicians serving the community, (ii) but also, and perhaps even more significantly, disparities with respect to other social determinants of health, such as crowded and/or sub-standard housing, poor quality education, lower income, inadequate nutrition, and higher risk work settings [3–5]. These factors, obtaining prior to the pandemic, account for the higher incidence of pre-existing conditions such chronic respiratory illnesses, obesity, diabetes mellitus, and hypertension.

The US is a dynamic society with major opportunities for advancement. Unfortunately, it is also burdened by a history of slavery and pervasive structural inequities that affect entire segments of the population [6]. Allocation guidelines are not responsible for the disparities within the US healthcare system or for the deeper and wider social inequities. Nevertheless, they tend to reflect and then perpetuate those inequities – thus putting people in historically discriminated groups in a kind of “double jeopardy.” *First,* people in these groups disproportionately suffer from health issues generated by disparities in respect to the social determinants of health. *Secondly,* given their higher frequency of comorbidities, they are more likely to be assigned lower priority for scarce life-saving resources, hence more likely to die from COVID-19.

A more just approach to resource allocation requires a wholesale mitigation of existing, long-term structural inequities, a reconsideration of allocation practices, and better pandemic preparedness. We examine the unintended consequences of applying traditional allocation schemas in a society plagued with racial and socioeconomic inequities, and present reflections on a new, more equitable paradigm that guarantees healthcare as a basic right for all.

## The impact of COVID-19 on minority communities

Throughout the pandemic in the US, ethnic minorities had the highest COVID-19 related attack, hospitalization, and mortality rates compared to the White population (Table 1) [7, 8]. A recent analysis has established a robust relationship between structural racism indices and level of disparity in the rates of COVID-19 mortality between Blacks and Whites for two thirds of the states. These correlations persist even after controlling for racial differences in comorbidities, the proportion of essential workers or those with high levels of exposure, and the number of underinsured or uninsured individuals [9]. In several communities across the US, age, comorbid conditions, and overall health status were major drivers of the higher incidence and mortality rates of COVID-19; predominance of racial/ethnic minorities, poverty, and lower educational attainment also had a strong impact on the disparate hospitalization and mortality rates due to COVID-19 [10, 11].

**Table 1:** The Impact of COVID-19 on Minority Communities

Risk for COVID-19 Infection, Hospitalization, and Death by Race/Ethnicity				
Rate Ratios Compared to White, Non-Hispanic persons	American Indian or Alaska Native, Non-Hispanic persons	Asian, Non-Hispanic persons^a^	Black or African American, Non-Hispanic persons	Hispanic or Latino persons
Cases	1.6x	0.7x	1.1x	2.0x
Hospitalization	3.5x	1.0x	2.8x	3.0x
Death	2.4x	1.0x	1.9x	2.3x

Table adapted from CDC data as of early November 2021 [7] (https://www.cdc.gov/coronavirus/2019-ncov/covid-data/investigations-discovery/hospitalization-death-by-race-ethnicity.html)

^a^Asian, Non-Hispanic persons represent a disparate and heterogeneous ethnic minority; the nuances and subtleties among several subgroups of Asian Americans (Indian and Taiwanese at the top of the income ladder vs. Japanese, Korean, Hmong, and Vietnamese in the middle or Nepalese and Burmese at the bottom) escape the raw data from the CDC [8]

Socioeconomic status and housing conditions also impacted the incidence of COVID-19 in the US. A significantly higher number of COVID cases was reported in zip codes with lower incomes and higher percentage of non-White residents [12]. To our knowledge, Indigenous Americans, Alaska Natives, Hispanics, and Blacks show no predisposing biologic characteristics to COVID-19 and SARS-CoV-2 has no specific predilection for them. Therefore, we argue that disparity in regard to the social determinants of health provides the best explanation for the disproportionate impact of COVID-19 in the US population [13].

In a study of Black and White Medicare recipients, the authors used simulated predictive models and attributed the higher hospitalization and mortality rates of Black patients with COVID-19 to the differences in the quality of hospitals to which they were admitted. However, they provided no data on hospital resources, particularly the ratio of patients to nurses or physicians, hospitals and ICU beds, number of hospital admissions, or independent measures of pre-pandemic hospital performance; and they did not adjust for the case load or for the stage or severity of COVID-19 at the time of admission across the hospitals [14]. We contend that the quality of care that is customary of the best and most resourceful hospitals can be easily degraded by a sudden and persistent surge of patients with COVID.

During a pandemic, the primary concern of municipal, state, and federal public health authorities is to save as many lives as possible, rather than to respect individual autonomy or achieve a more equitable society. Absent allocation guidelines, in the context of rapidly rising caseloads, healthcare professionals run the risk of applying traditional “first-come, first-served” models of care with disastrous results. To address acute maladies and shortages created by a pandemic, allocation guidelines have been developed by physicians, public health experts, social scientists, bioethicists, and others [15].

**Fig. 1:**
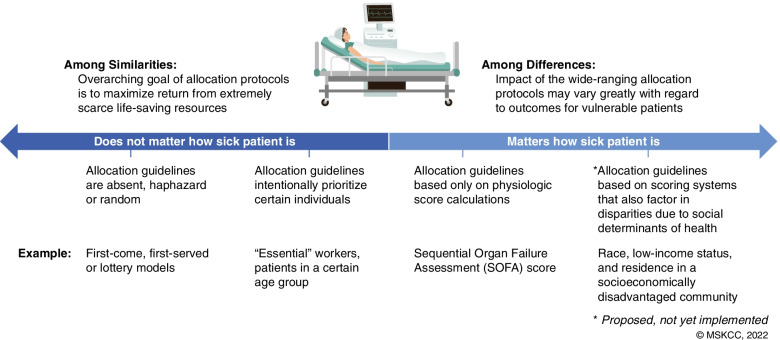
Illustration of the range of variation that can exist in allocation guidelines and practices

These schemes employ blunt, physiologically-based scoring calculations like the Sequential Organ Failure Assessment (SOFA) [16], thus prioritizing those (i) who are most likely to benefit from treatment measures; (ii) those who are in the best position to help save lives (for example, front-line healthcare workers, ambulance drivers, and others); and finally, (iii) those who serve in roles deemed ‘essential’ to a functioning society (members of government, truck drivers, and grocery workers). Allocation protocols share the noble ambitions of saving as many lives as possible, particularly in circumstances of extreme scarcity. However, their virtue is limited not only by the lack of consideration for social determinants of health but also by the state of equipoise related to the integration of physiologic scoring systems (Fig. 1).

Giving preference to groups of patients “most likely to benefit” based on physiologic scores is problematic because of the high prevalence of comorbidities in marginalized segments of the US population. Prioritizing frontline healthcare workers and other essential individuals because of their societal roles is in accordance both with utilitarianism and with Rawls’s Difference Principle [17]. But even this seemingly controversial prioritization is subject to some criticism because the roles in question – despite working to the advantage of all, at least “ex ante” – may not be as “open to all” on a fair opportunity basis, as “ideal” Rawlsian theory would require. Younger people may in general be prioritized by virtue not only of greater likelihood of benefiting from treatment (because of less co-morbidity, for instance) but also on account of both greater chances to live through several life’s stages and greater future life-expectancy [18]. However, because of disparities in social determinants of health, *even* younger people from historically disadvantaged groups, with higher rates of chronic ill-health and lower life-expectancy, may *not* fare as well under these allocation schemes.

Existing allocation models include the blueprint from the New York State Task Force for Life and the Law (NYSTF) [19], the University of Pittsburgh Medical Center’s (UPMC) “Allocation of Scarce Critical Care Resources During a Public Health Emergency”[16], merit-based systems, random distribution using simple lottery, and more complex schemes that consider or weigh various other attributes of individuals or groups of people [15]. For individuals whose initial suitability “scores” might be too close to warrant a rigid prioritization, it might become appropriate to bring in other factors to “break ties” – such as anticipated life-years gained from medical interventions, contributions to society, and marginalized status.

## The case against current allocation guidelines

**Fig. 2:**
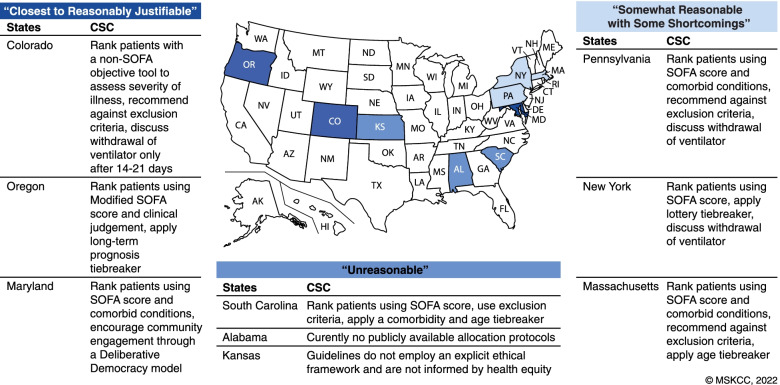
Various ventilator allocation guidelines across select US states

The US employs various approaches to allocation of scarce resources, as each state follows its own guidelines, if any [20]. As to the virtue and merits of the states that do have guidelines, the situation is complex and variable. We attempt to put this complexity in perspective through an assessment of a few guidelines as conveyed in Fig. 2. Maryland, for example, implements a Deliberative Democracy methodology that engages laypeople and healthcare professionals into informed discussions about how they would like to see their values reflected in policies [21]. Cooperation is foreseeably more likely when the development of allocation protocols is transparent and inclusive of prevailing public sentiments [21]. Colorado, Massachusetts, and Pennsylvania deliberately avoid the use of exclusion criteria because of the risk of discrimination against marginalized populations [20]. Alabama abandoned previous allocation policies that limited ventilator access to individuals with severe mental retardation, advanced dementia, or severe traumatic brain injury, without providing new guidelines [22] (Fig. 2).

Several provisions of allocation guidelines in New York and other states appeared to be inspired by the 1918 influenza pandemic and other viral outbreaks at the turn of the twenty-first century [23]. Reliance on historical precedents, particularly at the onset of the COVID-19 pandemic, may have led to erroneous empirical calculations or arbitrary clinical judgments, perhaps because of prognostic uncertainty and a lack of clinical data. As our understanding of the biology of SARS-CoV-2 improved, COVID-sensitive guidelines were proposed in Colorado, with deliberations about ventilator withdrawal after ~ 21 days of intubation [20].

In spite of Medicare, Medicaid, and the 2010 Affordable Care Act, ~ 30 million Americans lack insurance sufficient to cover prescribed treatments and recommended tests or avoid debt over medical bills [24]. In 2019, an estimated 14% of the US population was uninsured, with people of color more likely to be affected (Additional file 1: Appendix A) [25]. Many rural communities with disproportionately White Americans of low socioeconomic status similarly struggle as rural hospitals continue to close (Additional file 2: Appendix B) [26]. The elderly, the LGBTQ + community, and individuals who are disabled, homeless, incarcerated, or suffering major psychiatric illnesses also face limitations in access to medical care [27]. Besides food and hospital deserts, these injustices contribute in part to the higher incidence of comorbidities which in turn account for higher COVID-19 attack and mortality rates in certain segments of the US population.

These inequities have only become dramatically more evident during the COVID-19 pandemic. In mid-2020, Blacks, comprising 13% of the US population, made up to 30% of COVID-19 cases [28]. At several subsequent time points, the rate of infection among minority groups was higher than that of their White counterparts (Additional file 3: Appendix C) [7, 28]. Of all ethnic groups, Indigenous-Americans – with a long history of unjust treatment at the hands of the US government – have suffered the greatest rates of infection and mortality from COVID-19 [7, 29].

The UPMC and NYSTF frameworks claim that “race, ethnicity, gender, insurance coverage, and social worth” [16] do not influence allocation decisions. However, these guidelines do not adequately reflect today’s greater understanding of the role played by disparities regarding the social determinants of health. Thus, their proponents struggle in articulating a cogent argument of fairness in the setting of moderate or extreme scarcity and they acknowledge the inabilities of these allocation frameworks to mitigate “the disproportionate effects of COVID-19 on disadvantaged groups” in the US [30]. An alternative methodology of a “give back” scoring system – that shifts a 45-year-old Black man with COVID-19 and a low SOFA score from ineligibility to eligibility for ICU care after adjusting for markers of vulnerability (race, low income status, and residence in a socioeconomically disadvantaged community) – has been proposed [31]. Others have considered randomly allocating scarce resources through a lottery system, regardless of prognostic indicators [30].

We are not advocating for the eradication of allocation guidelines. Without them, we risk arbitrary and/or discriminatory decisions with tragic repercussions, as was often evidenced at various stages of the pandemic. As crisis standards of care were largely not deployed, many front-line responders were forced to rely solely on their clinical judgement in making prognostic predictions and critical decisions [32, 33]. At the hospital bedside, clinicians do not, and really cannot, introduce broader considerations about the social determinants of health; it is too late for that. But those factors significantly contribute to whether, and how, clinical care results in positive health outcomes.

## The case for a new framework

The ethical paradox of allocation cannot be addressed with a *solely* “technical” repair, such as improved pandemic preparedness with an increase in the production of critical medical resources. The solution also requires a renewed commitment to social justice and deeper respect for the inherent dignity of each person [34, 35]. To arrive at principles of justice, philosopher John Rawls proposes that decision-makers act behind a “veil of ignorance” (VOI) of citizens’ characteristics (such as financial status, race, or gender) in order to promote a social order that respects all individuals rather than one that is based upon personal circumstances [17, 36]. The COVID-19 pandemic prompts consideration of how transition to a more just society might be guided by VOI reasoning, only applied to the “non-ideal” background circumstances of historically generated and systemically embedded inequities. Studies suggest that VOI reasoning is most useful when people must make and justify decisions involving difficult trade-offs; observers are highly suspicious of decision-makers who rationalize their choices using utilitarian principles [36], but more accepting of otherwise uncomfortable compromises, if such solutions can be appreciated from a perspective (such as VOI) designed to give practical expression to their fundamental equality as persons.

### Healthcare reforms

In the US, healthcare costs continue to rise and far outpace other economically developed countries – but with lacking results. In 2019, US healthcare expenditures represented 17.7% of the US gross domestic product, compared with an average of 8.3% for European Union countries [37, 38]. Despite these outsized outlays, the US ranks only twenty-second in the world for healthcare outcomes [39]. Compounding this, data reveal that in the US, where income inequalities have risen to their highest level, healthcare outcomes by specific counties are significantly influenced by residents’ wealth [40]. Profound changes in our approach to the structural injustices that permeate the US healthcare system will lower the rates of infection and of mortality in currently marginalized communities for the inevitable next pandemic [41]. Disadvantaged groups should be afforded equal access to medical and other social goods that constitute the primary social determinants of health [42]. Consequentialist and other paradigms succeed in such nations as Spain, Italy, Iceland, Japan, and Norway, where social safety nets ensure universal healthcare access [43]. That such a lofty goal is imperative in the US, can and should be appreciated from both utilitarian and more deontologically justice-minded perspectives. Hence, there is hope that it can elicit strong support from an appropriately “overlapping consensus.”

In the US, an aversion to death and a determination to maintain life even at great cost make resource allocation more difficult [44]. More should be done to holistically address end-of-life planning, not for the sake of primarily saving scarce resources but rather to honor patient self-determination. During the COVID-19 outbreak, the Colorado Program for Patient-centered Decisions published information for patients about life-support and its physiological effects, duration, and ramifications [45]. When more fully informed about their options, adult patients of any age group with respiratory failure from severe COVID-19 may decide against a trial of mechanical ventilation. Pandemic preparedness requires attention to high-quality palliative care for patients who will die. These considerations should not be confused with age-discrimination. They are about respecting patient autonomy under some of the most difficult end-of-life circumstances [46, 47].

### Other critical reforms

Socioeconomic disparities which have so negatively impacted COVID-19 infection and mortality rates must be addressed [7, 12, 40]. Sub-standard housing conditions facilitate the spread of disease. Individuals with lower socioeconomic status tend to work in settings that facilitate contagion such as meat processing plants, supermarkets, and transit systems [48, 49]. These workers must be afforded PPE as the pandemic will render their work high-risk and turn them into significant vectors of disease transmission. Also, there must be substantial improvement in education, housing, and jobs for presently impoverished populations [50].

The overarching goal of allocation protocols is to maximize return out of extremely scarce life-saving resources. There is a limit to how far one can depart from the usual guidelines while deriving the full life-saving value of those resources. Indeed, in the absence of deeper societal reforms, both the standard schemes and the alternatives fall seriously short.

Deontological ethics set limits to the ways in which “consequences” may be made “optimal.” Recall the age-old aphorism: "the end doesn’t always justify the means.” One sort of deontological commitment might be to limit the ways in which a person can be “left aside” for the sake of maximizing net social utility. In “normal times,” we attend to the sickest patients first for admission, specialized care, and organ transplantations [51], but during a pandemic, we invert this practice and submit vulnerable patients to the risk of “double jeopardy” by favoring the most “salvageable.”

## Implementation of fair allocation guidelines

Crisis standards of care require coordination by public health authorities at city, state, and federal levels. The public’s trust in these authorities and healthcare institutions must be earned, especially among marginalized groups. Data are needed at county, state, and federal levels to assess the socioeconomic and health impacts of allocation practices and to calibrate the distribution of resources transparently and justly. For example, ambulances should deliver patients to hospitals with greater bed capacity [52] regardless of public or private status and of patient characteristics [12, 53], and PPE should be redeployed to medical centers with the greatest need. Community health centers could play an essential role in the outreach to disadvantaged neighborhoods and rural communities through health education, disease prevention, and the provision of clinical services, with the ultimate objective of mitigating comorbidities.

Improved healthcare access – along with other socio-economic reforms – may not altogether eliminate pandemic-related increases in rates of morbidity and mortality, but such measures are likely to make the effects of the next pandemic less severe than would have been otherwise. By addressing social determinants of health and by providing higher quality health education, we will be putting public health officials in a much better position to create more social cohesion and to motivate individuals to take more responsibility for their own health and the health of their families and communities. Our reflections are not without fault, but they represent a conscious effort to address and remediate the glaring disparities that have been magnified during the protracted COVID-19 pandemic.

## Conclusions

The COVID-19 pandemic experience has brought home to us, as perhaps never before, how important it is to deal with the underlying structural inequities. Traditional allocation guidelines strive to maximize net benefit for society in instances of moderate to extreme scarcity, but they fall far short of their goal unless inequities in socioeconomic status and healthcare access are significantly reduced. Otherwise, they inflict double jeopardy and disserve large swaths of the US population. Therefore, to be better prepared for the inevitable next pandemic, we must urgently begin two monumental undertakings. First, we must explore new allocation guidelines that incorporate social determinants of health. Second, we must address and reform the structural inequities in US society that account for the strikingly disparate COVID-19 attack, hospitalization, and mortality rates.

## Supplementary Information

**Additional file 1: Appendix A.** The Uninsured in America, by Race.

**Additional file 2: Appendix B.** The Uninsured in Rural America.

**Additional file 3: Appendix C.** Disproportionate COVID-19 Attack and Fatality Rates in Minority Populations Across the US.

## Data Availability

Not applicable.
