# Supplementary material for: COVID-19 double jeopardy: the overwhelming impact of the social determinants of health
Source: Int J Equity Health. 2022 May 24;21:76. doi: 10.1186/s12939-022-01629-0 (PMC9129892; doi:10.1186/s12939-022-01629-0)
Supplement: Supplementary file 1 — Additional file 1: Appendix A. The Uninsured in America, by Race. [file 12939_2022_1629_MOESM1_ESM.docx]

Appendix A: The Uninsured in America, by Race

<https://www.kff.org/racial-equity-and-health-policy/issue-brief/changes-in-health-coverage-by-race-and-ethnicity-since-the-aca-2010-2018/>

See: **Figure 1.** and **Figure 4.** *Uninsured Rates for the Nonelderly Population by Race and Ethnicity, 2010-2018*. Artiga, Samantha; Orgera, Kendal; Damico, Anthony. The Kaiser Family Foundation, 2020 (25).
