# Supplementary material for: COVID-19 double jeopardy: the overwhelming impact of the social determinants of health
Source: Int J Equity Health. 2022 May 24;21:76. doi: 10.1186/s12939-022-01629-0 (PMC9129892; doi:10.1186/s12939-022-01629-0)
Supplement: Supplementary file 2 — Additional file 2: Appendix B. The Uninsured in Rural America. [file 12939_2022_1629_MOESM2_ESM.docx]

Appendix B: The Uninsured in Rural America

<https://www.kff.org/wp-content/uploads/2013/01/the-uninsured-in-rural-america-update-pdf.pdf>

See: **Figure 1.** *Nonelderly Health Insurance Coverage by County Type, 1998.* and **Figure 2.** *Many Rural Workers Not Offered Employer-Sponsored Health Insurance.* The Kaiser Family Foundation, 2003 (26).
