# Supplementary material for: COVID-19 double jeopardy: the overwhelming impact of the social determinants of health
Source: Int J Equity Health. 2022 May 24;21:76. doi: 10.1186/s12939-022-01629-0 (PMC9129892; doi:10.1186/s12939-022-01629-0)
Supplement: Supplementary file 3 — Additional file 3: Appendix C. Disproportionate COVID-19 Attack and Fatality Rates in Minority Populations Across the US. [file 12939_2022_1629_MOESM3_ESM.docx]

Appendix C: Disproportionate COVID-19 Attack and Fatality Rates in Minority Populations Across the US

<https://www.motherjones.com/coronavirus-updates/2020/04/covid-19-has-infected-and-killed-black-people-at-alarming-rates-this-data-proves-it/>

See: **Figure 1.** *Infection rate for Black population with COVID-19,* **Figure 2.** *Fatalities in the Black community due to COVID-19,* and **Figure 3.** *Fatalities in the Latinx community due to COVID-19.* Rios, Edwin; Rangarajan, Sinduja. Mother Jones, 2020 (28).
